# Sudden cardiac death and kidney health related problems among Nepali migrant workers in Malaysia

**DOI:** 10.3126/nje.v9i3.25805

**Published:** 2019-09-30

**Authors:** Nirmal Aryal, Pramod R Regmi, Erwin Martinez Faller, Edwin van Teijlingen, Chan Chee Khoon, Adrian Pereira, Padam Simkhada

**Affiliations:** 1 Faculty of Health and Social Sciences, Bournemouth University, UK.; 2 Visiting Fellow, Datta Meghe Institute of Medical Sciences, India; Chitwan Medical College, Tribhuvan University, Nepal.; 3 Research Management Center, Tagum Doctors College, Philippines.; 4 Visiting Professor, Nobel College, Pokhara University, Nepal.; 5 Professor, University of Malaya, Kuala Lumpur, Malaysia.; 6 North South Initiative (NSI), Kuala Lumpur, Malaysia.; 7 Public Health Institute, Liverpool John Moores University, UK.; 8 Migrant Workers Health Alliance, Kuala Lumpur, Malaysia.

**Keywords:** Migrant workers, sudden cardiac deaths, kidney diseases, public patient involvement, labour migration

## Abstract

This paper reports on a consultation meeting that discussed two emerging health issues of Nepali migrant workers in Malaysia and the ways they can be addressed. Primarily, it focused on the issue of sudden cardiac deaths of Nepali migrant workers in Malaysia. This issue has been raised internationally by both scientific and media in the recent years. Secondly, it discussed kidney health related problem among Nepali migrant workers which has caught the attention of Nepali media recently. The meeting was organized in Kuala Lumpur, Malaysia on 19th April, 2019 where twenty people including health researchers, representatives of migrant related national and international organizations, and Nepali migrant workers participated. The meeting concluded that three types of data collection are needed: (1) good record of deaths, if at possible proper post-mortems; (2) a verbal autopsy tool to help identify underlying causes ; and qualitative research into kidney related problems.

## Introduction

Migration is a global phenomenon and particularly more common in low-and middle-income countries (LMICs). Around 3.4% of global population i.e. 258 million are international migrants in 2017, of which more than half (150.3 million) are migrant workers [1]. According to the World Bank report, international migrants sent a record high remittance of US$ 466 billion in 2017 which is three times higher than the official development assistance [2]. For the first time, migration is formally recognised in the 2030 Sustainable Development Goals (SDG) and has been acknowledged as a powerful tool for poverty reduction [3].

International labour migrant workers from Nepal have grown substantially in the recent decades. Mainly India, Malaysia and the six countries of Gulf Co-operation Council (GCC) are the key destinations [4, 5]. Ministry of Labour, Employment and Social Security has issued labour permits for 3.5 million Nepali in between 2008/09 to 2016/17, of which 86.4% were for Malaysia and the Middle East [5]. The economic contribution of Nepali migrants to the country is highly significant. In the fiscal year 2016/17, for example, they sent over US$6.1 billion in remittances which was 26.3% of Nepal’s total gross domestic product (GDP) [6].

Malaysia is the key destination for Nepali labour migrants. Of the total labour permits issued between 2008/09 and 2016/17, 29.9% were for Malaysia, followed by Qatar (21.6%) and Saudi Arabia (20.4%) [5]. On the other hand, Malaysia hosts around 2 million international migrants, mostly from Indonesia (35.3%), Bangladesh (28.4%), and Nepal (15.9%) [7]. International Organization for Migration (IOM) reports that currently there are around 2 to 4 million undocumented migrant workers in Malaysia [8]. In 2019, there are 3,27,529 Nepali migrant workers in Malaysia and 72.6% of them are working in manufacturing sectors [7]. Nepali are also the largest international migrant group working in manufacturing sectors in Malaysia. Including undocumented ones, experts estimate that there are at least half million Nepali migrant workers in Malaysia.

Cardiovascular related deaths (often sudden nocturnal deaths) among Nepali migrant workers is a particular concern in Malaysia [9] which are observed more frequently compared to other migrant groups in Malaysia. Recent studies among Nepali male migrants in the countries of Gulf Cooperation Council (GCC) and Malaysia document health vulnerabilities [10, 11], no previous study has established the cause of cardiovascular related deaths among Nepali migrants. Similarly, Nepali media frequently report on kidney health related risk in returnee migrant workers [12,13]. At this backdrop, Bournemouth University, UK collaborated with Malaysia based non-government organization (NGO) North-South Initiative (NSI) and Migrant Workers Health Alliance (MWHA) to discuss these issues with key stakeholders. On 19th April, a consultation meeting was organized in Kuala Lumpur which mainly aims to ascertain importance of these issues and to build consensus on further research plans if appropriate. Twenty participants including health researchers from local universities, international and national NGOs, Ministry of Health, migrant workers participated. Key viewpoints of the participants are given below.

### Sudden cardiac death of Nepali migrants

Nepali migrant workers, who are apparently healthy and without any symptoms of illness, are suddenly dying during their sleep. Interestingly, participants opine that the cases of these sudden nocturnal cardiac deaths are more frequently observed among Nepali migrant workers compared to other migrant groups which make this issue further intriguing. Most of these cases are reported as ‘cardiovascular deaths’ in a post-mortem report as a cause of death. Participants share their experience that usually comprehensive post-mortems are not performed and thus post-mortem reports do not include specific cause of death (for e.g., myocardial infarction, stroke for cardiovascular related deaths). In addition, health history of the dead migrant workers is also poorly available from any sources such as medical facilities, immigration offices, Nepali embassies, which further limits capacity to trace the cause of death. Police reports usually comprise a brief report with second-hand information.

Participating migrant workers said that Nepali migrants in Malaysia are mostly involved in manufacturing sectors which require physically demanding work with little opportunity of rest. Although normal daily working hour is 8 hours, it is normal to work overtime for an additional 8 hours in a day. Alcohol drinking is very common among Nepali migrant workers which participants believe could contribute to these sudden deaths. Many Nepali migrant workers also tend to consume cheap and locally brewed alcohol (often counterfeit version of popular brand) with high concentrations of methanol. Participants recalled that in 2018, 28 people including migrant workers from Myanmar, Bangladesh and Nepal died in Kuala Lumpur due to the alcohol poisoning.

Participants have generally agreed that the ‘verbal autopsy tool’ could be effective to identify causes of sudden cardiac deaths of Nepali migrant workers. They believe that administration of this tool to the close friends, room-mates, work supervisors or employers may provide personal health related, work related or any other information about the deceased. They emphasized on active involvement of Nepali embassy as it gets first-hand information on death of its nationals and has authority to represent them.

### Kidney health related issues of Nepali migrant workers

Participants seemed to be less aware of kidney health related problems among Nepali migrants in Malaysia. This may be because kidney health problems grow slowly and the symptoms are not seen at an early stage until disease is severe. However, in a separate discussion with Nepali Embassy in Malaysia, ambassador H.E. Mr. Udaya Raj Pandey informed that kidney health related problems are frequently observed in Nepali migrant workers.

Work related circumstances might precipitate kidney health risk in migrant workers. Nepali migrants work mostly on manufacturing sector where they usually work in a team or in a chain. If workers go to bathroom or break for drink, this working chain becomes disrupted and whole manufacturing process may stop. Malaysian manufacturing sectors are already crippled with labour shortage and they are not able (or willing) to recruit surplus workers. Thus, in many worksites, workers are not allowed (or need to inform supervisor) to leave the site except at the scheduled time. This means they have less opportunity for water intake and urination. One participating Nepali migrant worker who has been working in a Malaysia for more than a decade said that based on what he has seen drinking water is not available in at least half of the manufacturing sites. Many workers are exposed to heat stress generated by machineries or work process and are more likely to be dehydrated. This is apparent in other employment sector as well. Nepali migrants working as a security guard often complain that they are just allowed to use toilet twice a day because employers do not have replacement guard due to which they drink less amount of water. Due to physically exertive work with little rest and for many working for months without a day off, it is common among Nepali migrant workers to use painkillers regularly which is a risk factor for kidney health problems.

## Discussion and way forward:

A total of 5,982 Nepali migrant workers have died in 29 destination countries during the fiscal years 2008/2009 to 2016/17 [5]. Of these, the highest number of deaths (36%) occurred in Malaysia. This may be partly because Malaysia is the main destination country for the Nepali migrant workers. According to the report of Ministry of Labour and Employment (Nepal), 1,562 Nepali have died in Malaysia during 2008/09 to 2014/15, of which 423 (27.1%) were reported to have cardiac arrest or heart attack, including during the sleep [5]. Cardiovascular related deaths, based on post-mortem reports, may be the leading cause of death among Nepali migrant workers in Malaysia in 2015 (22.3%) [9]. The aforementioned ministry report has acknowledged under-reporting of number of death as it is based on compensation claim application after the death and the figure from the embassy was higher for all destination countries.

There is some evidence of an association between sudden cardiac death and the risk factors experienced by the migrant workers; for example long working hours [14], exposure to extreme temperature [15], strenuous physical activity [16], heavy alcohol drinking [17], being overweight [18] etc. This is further underpinned by the report that factory workers/production operators accounted for 56% of total cardiovascular related deaths among Nepali migrant workers in Malaysia in 2015 [9].

Nepal does not have a comprehensive record of age- and sex- specific cause of deaths of its population to compare the CVD related mortality rate with migrant workers. However, Nepali migrant workers are mostly young (under 30 years) and have to undertake mandatory medical examination during pre-departure in Nepal and post-arrival period in a destination country. Hence, they can be considered as a healthy group who are less likely to have developed cardiovascular risk factors at a level triggering death.

Overall, the consensus was made in the meeting that lack of comprehensive post-mortem report was a major problem for identifying the causes of these sudden cardiac deaths of Nepali migrant workers. It is imperative to collect, record and publish accurate causes of these deaths [19]. Participants unanimously agreed that development and implementation of a verbal autopsy tool may help to identify these causes in the current situation. It will be useful to plan public health intervention even when the detailed post-mortem report is available. Further immediate action plan includes: a developing network and building research team including health researchers and clinical pathologists from both Nepal and Malaysia as well as migrant workers, Nepali embassy and Ministry of Health, Malaysia.

With regards to kidney health related problems, participants suggested that qualitative studies would be useful at the initial stage. This may include carrying out research among Nepali migrant workers with kidney health problems in Malaysia as well as among returnee migrant workers from Malaysia who are under the treatment of kidney health related problems in Nepal.
